# Murine model of hepatic breast cancer

**DOI:** 10.1016/j.bbrep.2016.07.021

**Published:** 2016-08-04

**Authors:** Rishi Rikhi, Elizabeth M. Wilson, Olivier Deas, Matthew N. Svalina, John Bial, Atiya Mansoor, Stefano Cairo, Charles Keller

**Affiliations:** a Children’s Cancer Therapy Development Institute, Fort Collins, CO 80524, USA; b Yecuris Corp., Tigard, OR 97062, USA; c XenTech, 91000 Evry, France; d Papé Family Pediatric Research Institute, Department of Pediatrics, Oregon Health & Science University, Portland, OR 97239, USA; e Department of Pathology, Oregon Health & Science University, Portland, OR 97239, USA

**Keywords:** HER2, human epidermal growth factor receptor 2, FRG™ KO, [ Fah(-/-) R ag2(-/-)Il2r g (-/-)]), NOD, Non-obese diabetic, Ad:uPA, Adenovirus Urokinase Plasminogen Activator, pfu, plaque forming units, DMEM, Dulbecco's Modified Eagle Medium, HCM, Hepatocyte Culture Medium, IACUC, Institutional Animal Care and Use Committee, AFP, Alpha Fetal Protein, Hep Par 1, Hepatocyte Paraffin 1, GPC3, Glypican-3, Breast cancer, Preclinical model, Liver metastasis

## Abstract

**Background and aims:**

Breast cancer is the most common cancer in women and the second leading cause of cancer-related deaths in this population. Breast cancer related deaths have declined due to screening and adjuvant therapies, yet a driving clinical need exists to better understand the cause of the deadliest aspect of breast cancer, metastatic disease. Breast cancer metastasizes to several distant organs, the liver being the third most common site. To date, very few murine models of hepatic breast cancer exist.

**Methods:**

In this study, a novel murine model of liver breast cancer using the MDA-MB-231 cell line is introduced as an experimental (preclinical) model.

**Results:**

Histological typing revealed consistent hepatic breast cancer tumor foci. Common features of the murine model were vascular invasion, lung metastasis and peritoneal seeding.

**Conclusions:**

The novel murine model of hepatic breast cancer established in this study provides a tool to be used to investigate mechanisms of hepatic metastasis and to test potential therapeutic interventions.

## Highlights

• We present a novel murine model of hepatic breast cancer.
• Histomorphology is uniform and consistent with the human disease.
• This model can be used to test preclinical therapeutic regimens.

## 1 Introduction

Breast cancer is the most common cancer in women and the second leading cause of cancer-related deaths in this population. It is estimated that there will be 231,840 new cases of breast cancer and 40,290 breast cancer related deaths in women in 2015 [1]. The survival rate for patients diagnosed with breast cancer is a function of a variety of factors, the most important being metastasis. In fact, the survival rate for patients diagnosed with metastatic breast cancer to distant organs falls from 99% to 25% when compared to primary site-only cases [1]. Although 61% of breast cancers are localized, 10–15% of patients develop metastasis to distant organs [1], [2]. Predicting the risk of metastasis is often difficult due to a lack of biological markers and the heterogeneous nature of breast cancer [2]. Histological typing does not provide tremendous predictive value for metastasis, yet invasive ductal carcinoma is the most common invasive breast carcinoma and has one of the lowest survival rates [2]. At a molecular level, HER2-enriched and triple-negative (estrogen receptor negative, progesterone receptor negative and HER2 negative) tumors are associated with a higher rate of liver metastases [3]. The fact that metastasis does not respond well to traditional management techniques of surgery, radiation and chemotherapy is a key motivator to developing better research models [2], [4].

Murine models are a useful tool for researchers and oncologists in characterizing and understanding the spread of breast cancer. A summary of these models is presented in Table 1. Unto now, a model of primarily hepatic metastasis is lacking. In this study, we report the development of a novel murine model of hepatic breast cancer using the breast cancer cell line MDA-MB-231, a triple-negative adenocarcinoma cell line derived from a 51-year-old Caucasian female [5].

**Table 1:** Literature review of murine models of hepatic breast cancer. The table states the cell lines for various murine models of breast cancer hepatic metastasis as well as the purpose of each study.

**Cell Line**	**Mouse**	**Inoculation site**	**Liver met?**	**Other met?**	**Study purpose**	**Reference**
MDA-MB-435	Female nude	Mammary fat pad	Yes	Yes	Use of surgical orthotopic implantation	[7]
MDA-MB-435	Female athymic NU/NU	Right second mammary gland	Yes	Yes	Optical imaging	[8]
4T1	Female BALB/c	Mammary fat pad	Yes	Yes	Effect of bisphosphonate	[9]
4T1	Female BALB/c	Mammary fat pad	Yes	Yes	Effect of factor 4	[10]
4T1	Female BALB/c	Mammary fat pad	Yes	Yes	Use of dabigatran	[11]
4T1	Female BALB/c	Mammary fat pad	Yes	Yes	Effect of R428	[12]
4T1	BALB/cfC3H	Mammary fat pad	Yes	Yes	Imaging	[13]
4T1/luc	BALB/c	Mammary fat pad	Yes	Yes	Effect of zoledronic acid	[14]
MDA-MB-231	Female NCr nu/nu	Mammary fat pad	Yes	Yes	Effect of pro-matrix metalloproteinase-2	[15]
MDA-MB-231	Female athymic nude	Mammary fat pad	Yes	Yes	Effect of GLV-1h153 in TNBC	[16]
MDA-MB-231	BALB/cAnN.CG-Foxn1 nu/CrINar	Tail vein	Yes	No	Effect of VP1	[17]
MDA-MB-231		Mammary pat	Yes	Yes	Effect of PEDF	[18]
MDA-MB-231	Female nude	Mammary fat pad	Yes	Yes	Effect of CH50	[19]
MDA-MB-231/luc	Female Rag2-/-II2rg-/-	Mammary fat pad	Yes	Yes	Effect of phenytoin on TNBC	[20]
4TLM	BALB-c	Right upper mammary gland	Yes	Yes	Multi metastatic model	[21]
4THM	BALB-c	Second right chest mammary pad	Yes	Yes	Effect of semapimod	[22]

## 2 Materials and methods

### 2.1 Preconditioning with Ad:uPA

Cesium chloride banded and plaque assay titered Ad: uPA was diluted to 1.25×10^9^ plaque forming units (pfu)/100 µL in sterile 0.9% saline and filtered using an Acrodisc syringe filter with 0.45 µM HT Tuffryn membrane. Each mouse was anesthetized using isoflurane and dosed via retro-orbital vein injection with 1.25×10^9^ pfu per 25 g of body weight.

### 2.2 Transplant with human MDA-MB-231 cells

Cryopreserved human MDA-MB-231 cells (Xentech, France) were thawed and expanded in Advanced DMEM with 10% fetal bovine serum and antibiotics. After expansion, the cells were detached from the tissue culture plate by treatment with TryPLE, collected, and centrifuged at 150×*g* for 10 min at 4 °C. The MDA-MB-231 cells were resuspended in HCM (catalog# CC-3198, Lonza, Basel, Switzerland) to a concentration of 1–2×10^6^ cell/mL and diluted 1:1 in 0.4% trypan blue; the cell number and viability were determined using a hemocytometer. MDA-MB-231 cells were centrifuged again at 150×*g* for 10 min at 4 °C and reconstituted in HCM (Lonza) at 10×10^6^ cells/mL. To counteract rapid vascular clearing and allow adequate time for liver engraftment, the MDA-MB-23 cells were then mixed 1:1 with Matrigel phenol red free (cat#356237, BD Biosciences, San Jose, CA); this generated a 100 µL transplant solution. The mixture was kept on ice to prevent the Matrigel phenol red free from solidifying.

All animal studies were done with approval of the IACUC for Yecuris, Inc. FRG™ KO/NOD mice were anesthetized using isoflurane. Hair was removed from the lateral region and mid-line of each animal using Nair® hair removal cream, Then the 100 µL of transplant solution was delivered through the dermal layer into the frontal lobe of the liver 22–24 h after pretreatment with Ad:uPA. The mice were observed each day post-transplant and were provided water and food ad libitum. The transplanted mice were placed on the standard Nitisinone cycling for all xenografted mice.

### 2.3 Human AFP ELISA

Human alpha-1 fetoprotein (AFP) ELISA kit (cat#ab108838) was obtained from Abcam (Cambridge, MA). Sera samples were collected at four weeks post-transplantation and stored at −80 °C until assayed. The human AFP levels from each mouse were assayed using 50 µL of sample at a 1:5, 1:20 or 1:100 dilution of sera according to the manufacturer's protocol.

### 2.4 Immunostaining

Immunohistochemical staining was performed on formalin-fixed, paraffin-embedded tissue sections. Four micron sections were prepared on charged slides. Staining was performed on Ventana XT automated instruments (Ventana, Tucson, AZ) with ultra-view polymer-based DAB detection system. Clone designations, working dilutions, and sources for the commercially available antibodies were as follows: AFP (Clone A0008, dilution 1:3000, Dako, Carpinteria, CA), Hep Par 1 (Clone M7158, dilution, 1:250, Dako), Glypican 3 (Clone 790-4564, predilute, Ventana, Tucson, AZ), Her 2-neu (Clone 790-2991, predilute, Ventana), p53 (Clone 790-2912, predilute, Ventana), Ki-57 (Clone 790-4286, predilute, Ventana) Sections were counterstained with hematoxylin. Appropriate positive and negative controls were present in every case. Negative controls were performed by replacing the primary antibody with normal mouse serum. Final interpretation of the staining results was performed by a surgical pathologist using light microscopy.

## 3 Results

**Fig. 1:**
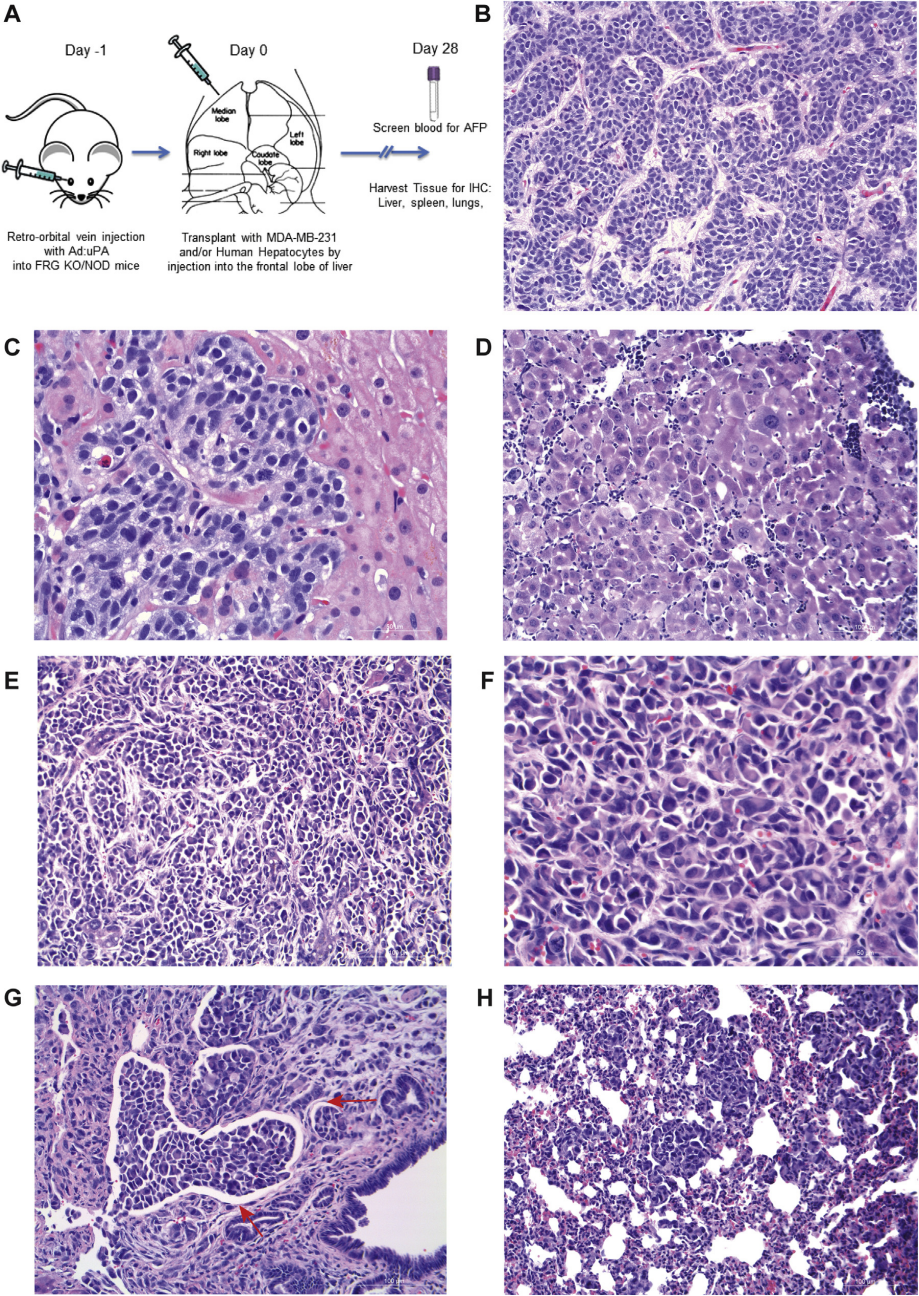
Murine model of hepatic breast cancer (A) Diagram of murine engraftment. (B, C) Photomicrograph of liver metastasis in a human patient. (D) Murine tumor of almost exclusively an undifferentiated cell component. (E, F) Additional histological features of the murine model. (G) Vascular invasion was a prominent (depicted with red arrows), and (H) lung metastasis was a consistent feature. Peritoneal seeding was common but no intrasplenic disease was seen (data now shown).

**Fig. 2:**
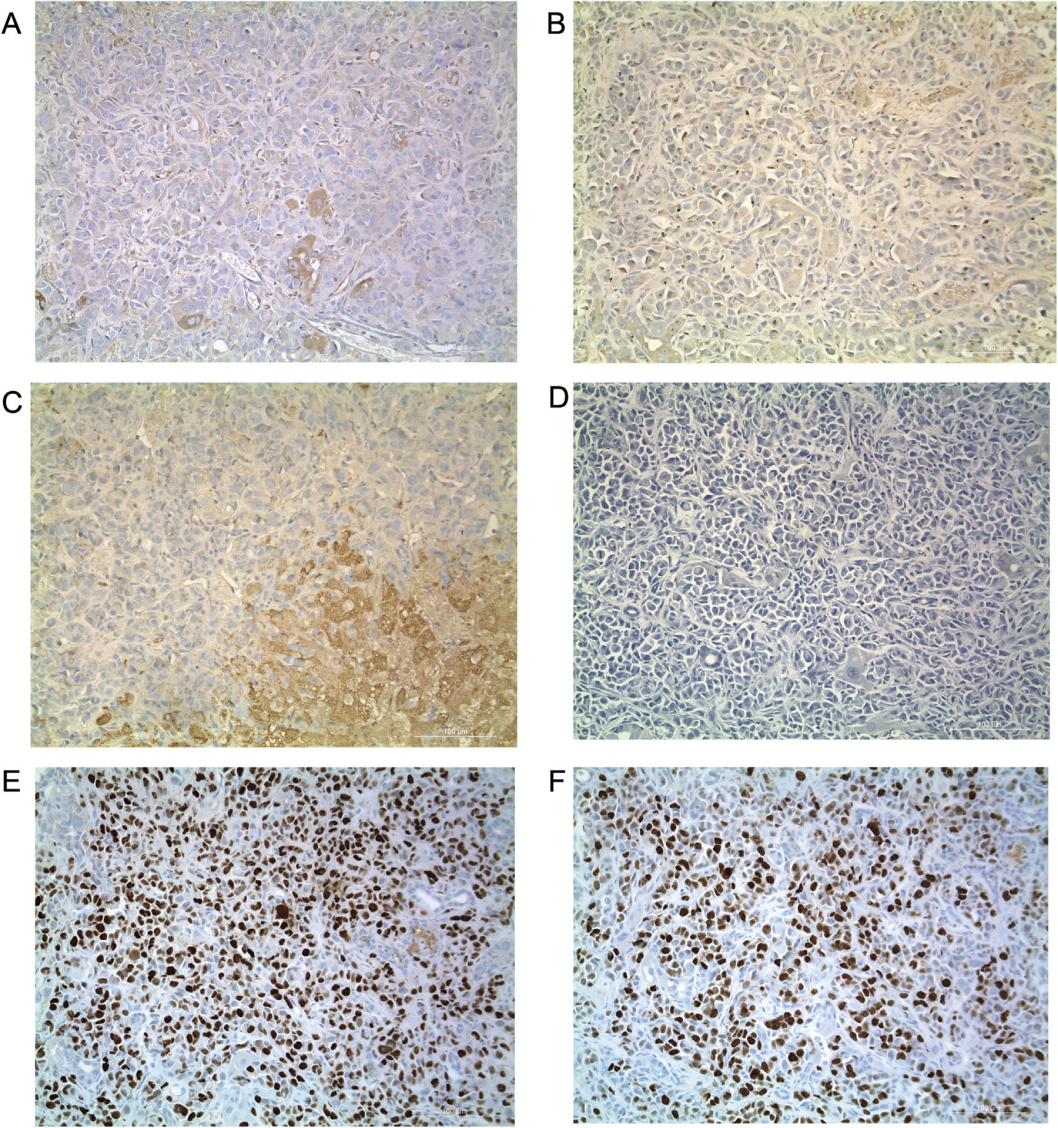
Immunostains for intrinsic hepatic and breast cancer markers (A) Intrinsic liver marker AFP. Serum AFP by ELISA was below the limit of detection (data not shown). (B) Liver marker Glypican-3. (C) Liver marker Hep Par 1. (D) Cancer marker HER2/Neu. (E) Cancer marker P53. (F) Cancer marker Ki-67 (nuclear).

To create a murine model of hepatic breast cancer, we preformed direct injection of MDA-MB-231 cells into the frontal lobe of the liver (Fig. 1A) of mice previously shown to have a capacity to be repopulated with primary human hepatocytes. This FRG™ KO [ Fah(-/-) R ag2(-/-)Il2r g (-/-)]) on the NOD mouse strain has been previously described [6]. In this model, however, co-injection of primary human hepatocytes offered no additional benefit to engraftment, but only increased the degree of necrosis (data not shown). Therefore analysis was restricted to FRG™ KO/NOD mice injected with MDA-MB-231 tumor cells only. Histological typing revealed exclusively breast cancer cells (and not murine hepatic tumors). Characteristic human breast cancer liver metastases are shown in Fig. 1B and Fig. 1C. The murine model approximated this histology (Fig 1D-1F). Common features of the model were vascular invasion (Fig. 1G), lung metastasis (Fig. 1H) and peritoneal seeding (not shown). There was no splenic disease seen in the model. The immunostains for intrinsic liver cancer markers AFP, Hep Par 1 and GPC3 were all negative (Fig. 2). The immunostain for Her-2 neu were negative, and immunostain for p53 was positive in 80% of tumor cells and the Ki-67 proliferation index was 60% (Fig. 2). An ELISA was done in order to measure serum AFP, but the level was below the limit of detection.

## 4 Discussion

A strong clinical need exists to better understand breast cancer metastasis. The novel murine model of hepatic breast cancer established in this study provides a tool to be used to investigate mechanisms of hepatic metastasis and potential therapeutic interventions. Characteristics of this murine model are consistent with clinical features of breast cancer metastasis, such as vascular invasion and metastasis to the lung (the second most common organ affected by breast cancer metastasis) [2]. Furthermore, tumors in these FRG™ KO/NOD mice cannot be attributed to strain background, as the tumor markers in this model were not consistent with primary hepatic tumors. While a limitation of this model was that it was not generated with a HER2+ cell line, future studies may include this biological feature. However, the tumor samples were positive for well-established breast cancer cell markers p53 and Ki-67. It is also important to note that there was no control for the FAH phenotype in this experiment. Future studies will investigate the necessity and level of benefit of the FAH phenotype in engraftment. In summary, however, the procedure reported here represents a reproducible way to generate a hepatic breast cancer preclinical model to understand tumor-microenvironment interactions and to test preclinical therapeutic regimens.
